# Implementation and perceived effectiveness of gun violence restraining orders in California: A qualitative evaluation

**DOI:** 10.1371/journal.pone.0258547

**Published:** 2021-10-19

**Authors:** Veronica A. Pear, Julia P. Schleimer, Elizabeth Tomsich, Rocco Pallin, Amanda Charbonneau, Garen J. Wintemute, Christopher E. Knoepke

**Affiliations:** 1 Department of Emergency Medicine, Violence Prevention Research Program, UC Davis School of Medicine, Sacramento, California, United States of America; 2 RAND Corporation, Santa Monica, California, United States of America; 3 Division of Cardiology, University of Colorado School of Medicine, Aurora, Colorado, United States of America; 4 Adult and Child Consortium for Outcomes Research and Delivery Science, University of Colorado School of Medicine, Aurora, Colorado, United States of America; University of Connecticut, UNITED STATES

## Abstract

**Background:**

Uptake of gun violence restraining orders (GVROs), which temporarily prohibit the possession and purchase of firearms and ammunition from individuals at particularly high risk of harming themselves or others with a firearm, has been slow and heterogenous across California. Insights into the implementation process and perceived effectiveness of the law could guide implementation in California and the many states that have enacted or are considering enacting such a law.

**Methods:**

We conducted 21 semi-structured interviews with 27 key informants, including judges, law enforcement officers, city and district attorneys, policy experts, and firearm violence researchers. Analysis of transcripts was guided by grounded theory and the Consolidated Framework for Implementation Research (CFIR).

**Findings:**

The following constructs emerged within 4 CFIR domains as salient features of implementation: 1) implementation characteristics: risk of violence, cost, and adaptability; 2) outer setting: interagency coordination and local firearm ideology; 3) inner setting: readiness for implementation and law enforcement firearm culture; and 4) implementation process: planning and engaging with those involved in implementation. Key informants perceived the law to be effective, particularly for preventing firearm suicide, but agreed that more research was needed. While most indicated that the law resulted in positive outcomes, concerns about the potential for class- and race-based inequities were also raised.

**Conclusions:**

Implementation of the GVRO law in California was hampered by a lack of funding to support local proactive implementation efforts. This resulted in ad hoc policies and procedures, leading to inconsistent practices and widespread confusion among those responsible for implementation. In states that have not begun implementation, we recommend dedicating funding for implementation and creating local procedures statewide prior to the law’s rollout. In California, recommendations include providing training on the GVRO law—including an explication of agency-specific roles, responsibilities, and procedures—to officers, city attorneys, and civil court judges.

## Introduction

Extreme risk protection orders (ERPOs), called gun violence restraining orders (GVROs) in California, temporarily prohibit the purchase and possession of firearms and ammunition from individuals at high risk of harming themselves or others with a firearm but not otherwise prohibited from firearm ownership. They are an important example of a risk-based approach to firearm violence prevention, in which interventions focus on individual persons rather than on the general population. ERPO policies represent actions that can be taken by individual states at a time when attempts to strengthen federal firearm violence prevention policies have been largely unsuccessful. They enjoy substantial public support, including among firearm owners [1, 2].

**Fig 1:**
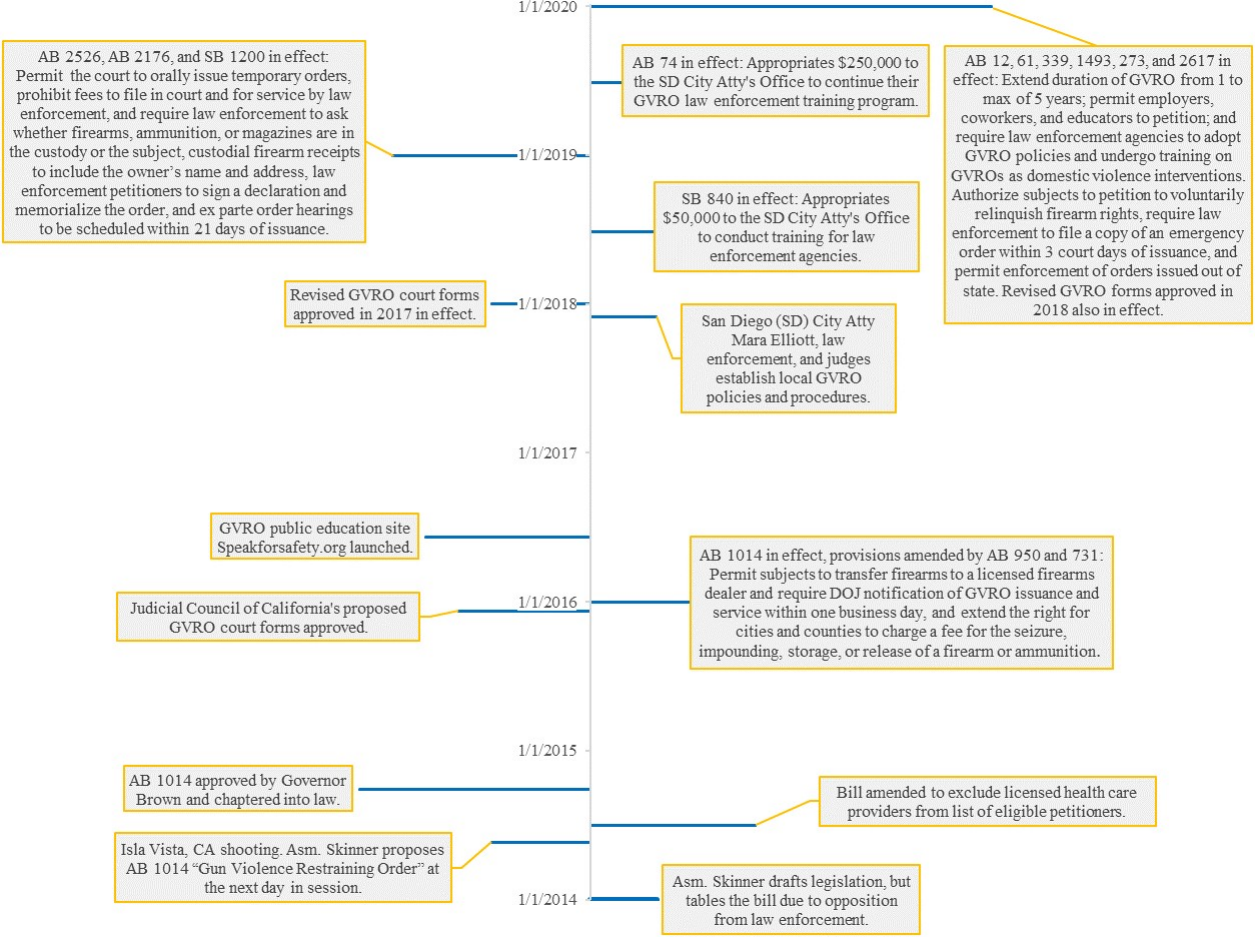
Timeline of the GVRO law and implementation efforts.

In 2016, California became the first state to implement such a law, allowing law enforcement, family members, and, beginning September 2020, employers, coworkers, teachers, and school administrators, to petition the court for a GVRO (see Box 1 for process details and Fig 1 for a timeline of key events) [3]. Many states followed suit; as of July 2021, ERPOs have been adopted by 17 states and the District of Columbia [4]. Permissible petitioners vary by state, but most allow for law enforcement officers and family or household members to petition.

**Box 1. Overview of the GVRO process**

**Petition:** All GVROs begin with an inciting event in which a person who owns or is trying to acquire a firearm threatens to harm others or themselves. This is followed by a petition to the court for either an emergency or a temporary GVRO, which generally last for up to 21 days (until a hearing can be held) if granted. Only law enforcement officers (LEOs) can petition for an emergency order, which is typically granted by a judge over the phone while the officer is in the field. LEOs, immediate family members, and household members can petition for an *ex parte* temporary order, which is granted by a judge during normal business hours. As of September 2020, the law expanded to allow employers, coworkers, teachers, and school administrators to serve as petitioners for temporary GVROs (see Fig 1 for a timeline of key events).

**Service:** After a judge grants an emergency or temporary GVRO, it is served to the respondent (the subject of the order) by a LEO, a process server, or another adult. LEOs are required to request the immediate relinquishment of firearms by GVRO respondents upon service, but if the respondent refuses, they are not authorized to seize firearms without a search warrant unless the firearms are in plain sight. If LEOs do not request immediate relinquishment or if the order is served by someone other than an officer, the respondent has 24 hours to comply with the order and 48 hours to provide proof to the court that they transferred all firearms to a licensed firearm dealer or law enforcement agency.

**Hearing:** Subsequent to issuance of an emergency or temporary order, a hearing is scheduled in order for a judge to determine whether there is sufficient evidence of ongoing risk to extend the order for 1 year (or up to 5 years, beginning in September 2020), called an “order after a hearing.” Respondents may petition for the order to be terminated once every year that the order is in place. If a petitioner still considers the respondent a danger to others or themselves within 3 months of when the order is to expire, a petitioner can ask the court for a renewal.

**Expiration:** Once an order has expired, if a respondent wants to regain custody of their firearms, they may submit an application to the California Department of Justice to obtain a letter of eligibility affirming they are no longer prohibited, which is contingent upon passing a background check for firearm ownership. The respondent must then present their letter of eligibility to the law enforcement agency or retailer to whom firearms have been temporarily transferred for safekeeping.

Similar warrant-based extreme risk laws have existed in Connecticut and Indiana since 1999 and 2005, respectively, which are more limited than ERPOs in that they do not prevent unarmed individuals from purchasing firearms and can only be sought by law enforcement officers. These laws appear to be effective at preventing firearm suicide [5–7]. While the effectiveness ERPO laws have against other forms of firearm violence have yet to be rigorously evaluated, we found the law in California to have promise: in 21 cases, GVROs were issued against individuals threatening mass shootings, none of which were carried out [8]. However, the potential impact of GVROs in California may be stifled by slow and uneven uptake [9].

In the first 4 years of policy implementation (2016–2019), there were 1,094 GVRO respondents in California [9], the majority of whom were male and white. During this time, there was substantial geographic variation in GVRO use. Santa Clara county and counties in Southern California issued the most GVROs. San Diego county alone accounted for over 30% of GVRO respondents (355 of 1,094), while 14 counties did not issue any GVROs. Statewide, GVRO use increased over time, with minimal use until 2018. Preliminary data suggest that this trend has continued through 2020 [10]. Understanding how and why GVRO implementation varies, and what practitioners perceive as the impact and effectiveness of GVROs, can inform implementation strategies in California and beyond.

Very little is known about barriers to and facilitators of ERPO implementation. Studies of risk warrants in Connecticut and Marion County, Indiana included findings from a small number of key informant interviews (11 and 5, respectively) suggesting implementation was hampered by issues such as understaffed police departments and a lack of policies, procedures, and training [5, 6]. Similarly, a report on ERPOs in Broward County, Florida (where only law enforcement officers are permitted to petition) noted that establishing local protocols was key to successful implementation [11]. There is a need to evaluate implementation of these policies more thoroughly, as many states are just starting their implementation efforts. Moreover, generalizability of these past findings to California is questionable because of the differences between the policy in these states as well as the different sociopolitical contexts in which implementation is being carried out.

To examine how California’s GVRO law is being implemented, we conducted semi-structured interviews with 27 key informants representing law enforcement, city and district attorneys (prosecutors), judges, and experts in firearm violence policy. We sought to elucidate process-based and contextual factors that encouraged or hindered the uptake or perceived effectiveness of these orders and to describe key informants’ perceptions of GVRO outcomes and effectiveness. This is the first qualitative study of GVRO implementation in California. Findings will be of interest to policymakers, professionals involved in ERPO implementation, and violence prevention researchers in California and other states that have adopted or are considering adopting such a policy.

## Materials and methods

### Study sample

Potential key informants were selected due to their experience with or demonstrated knowledge of GVROs (e.g., through published reports). They were identified through professional relationships with the authors, activity in the gun violence prevention community, public records indicating involvement in the service or disposition of GVROs, and by recommendation from other informants. Participants were initially selected with purposive heterogenous sampling (according to professional roles and geography) to capture variation in their experiences [12]. Subsequent participants were selected with theoretical sampling, determined by the need to further explicate components of the developing theory [13].

From May 22, 2019 to April 24, 2020, trained, experienced interviewers (JPS, CEK, Shani Buggs and Brian Hayes) conducted semi-structured telephone interviews with key informants, each lasting around 50 minutes (range: 23–76 minutes). Participants provided verbal informed consent prior to interviews. They were asked about their experience with and perceptions of the GVRO law, including its implementation process and effectiveness at reducing firearm violence. After reaching theoretic saturation, we identified and interviewed 2 additional key informants, specifically selected to maximize diversity with respect to professional role and geography to ensure our theory captured the widest possible range of experiences [14].

### Interview protocols

The interview protocol for key informants directly involved in enforcement differed from protocol for policy experts, the researcher, and advocates (see S1 Appendix for complete protocols). We asked all key informants about their knowledge of the policy (e.g., *For what kinds of cases do you think GVROs are intended*? *For which do you think they are most suitable*?), their professional experience with GVROs or the policy, and their opinion of the GVRO policy (e.g., *How much would you agree with the following statement…[O]ther states should adopt GVRO policies*). However, we only asked officers, attorneys, and judges detailed questions about GVRO enforcement (e.g., *[H]ow frequently*, *when firearm and ammunition recovery was the objective*, *was recovery achieved*?). A few interview questions were tailored specifically to the role of the practitioner (e.g., For judges: *Have you ever denied a petition*? *What were the specifics of the case and reasons for denial*? For law enforcement officers regarding a denied petition: *Did you feel the denial was justified*?). Instead of questions about GVRO cases, we asked policy experts, the researcher, and advocates more detailed questions about the GVRO law (e.g., *What challenges*, *if any*, *does the GVRO law currently face*?).

Audio recordings from key informant interviews were professionally transcribed, and transcriptions were uploaded into Dedoose (version 8.3) to facilitate team-based qualitative analysis.

### Analytic approach

We took a grounded theory approach with constant comparative methods to simultaneously create a theory describing the dissemination and implementation of GVROs across California and to ensure that theoretic domains influencing implementation were applied consistently [15]. Members of the analytic team met regularly to sort and synthesize data [13], including the creation of new codes and the strategic collection of new data. To maximize clarity and comparability with other implementation research, we used the Consolidated Framework for Implementation Research (CFIR), a typology for implementation science intended to promote comparability of findings from different contexts by organizing themes within 4 interacting domains: intervention characteristics, outer setting, inner setting, and process [16]. The domains offer a way of organizing findings into broad categories, which are then further organized into sub-domain constructs. Intervention characteristics are features of the intervention that influence implementation. Outer setting refers to the larger sociopolitical environment in which the implementing bodies reside, and inner setting refers to the contexts within the implementing bodies themselves. Finally, process refers to the implementation process. Additional constructs beyond those suggested by CFIR were added within domains as needed.

Coding and theme construction were conducted using a mixed inductive-deductive method developed and used previously by members of the research team [17–19]. In this process, an initial *a priori* codebook was developed with specific codes addressing the overarching project goals (i.e. to describe the implementation process as it is understood by various stakeholders in California). This codebook was then further refined and expanded reflexively after interviews were complete but before coding had commenced. Coders included a group of six investigators (VAP, JPS, ET, RP, AC, CEK), with both masters- and doctoral-level training in public health, violence prevention, qualitative methods, and health services research, and included those who conducted the majority of interviews. Coding included group-based open coding of 6 interviews, with the entire analytic team meeting regularly to compare notes and consolidate codes. This process refined the final codebook, which was then applied to the remaining interviews. Further, the iterative nature of code development is designed to directly influence the construction of themes, as initial codes are geared toward research questions and position CFIR as an organizational and explanatory framework. These are then refined as 1) interviewers and coders gain immersion in the data and 2) coders consolidate coding definitions collectively, encouraging the continuous emergence of both inductive (ad hoc) codes and constructed thematic understanding. We utilized team coding and investigator triangulation to maximize reliability, confirmability, and contextual authenticity of findings [20, 21]. Grounded theory itself also increases confirmability by rooting the theory in the participants’ own words, and the multidisciplinary approach ensures that no viewpoint dominated data interpretation. Confirming and disconfirming cases were discussed within the entire analytic group until thematic consensus was reached. Thematic saturation, the point at which additional interviews were providing little to no new thematic detail, was also determined as a group. To the greatest extent possible, we followed the consolidated criteria for reporting qualitative studies (COREQ-2) recommendations [22].

This study was approved by the Institutional Review Board at the University of California, Davis.

## Results

We conducted 21 interviews involving 27 key informants. At participants’ request, 4 interviews involved 2–3 key informants who were coworkers. All but 7 individuals (3 interviews) consented to being recorded. In these cases, notes taken by interviewers during and immediately following the conversation were used to supplement analyses. Key informants comprised 7 judges, 7 law enforcement officers (LEOs), 6 deputy city or district attorneys, 4 policy experts, 2 firearm violence prevention advocates, and 1 academic researcher. Of the 14 California counties that had issued at least 1 GVRO per year by the start of 2020 [9], 9 were represented, mostly in Southern California and the Bay Area (Table 1). Key informants worked in counties in the fourth (Alameda, Los Angeles, Sacramento, San Joaquin, San Diego, Santa Clara), third (Contra Costa, San Francisco), and second (Nevada) quartiles of GVRO uptake between 2016 and 2019. Correspondingly, key informant counties accounted for the fourth (Alameda, Los Angeles, Sacramento, San Joaquin, Santa Clara, San Diego) and third (San Francisco) quartiles for total number of firearm deaths by assault or suicide between 2016 and 2019 [23]. Overall, counties with higher counts of firearm deaths issued higher numbers of GVROs.

**Table 1:** Key informant characteristics.

Characteristic	Key Informants (n = 27)
Role vis-à-vis GVROs, N (%)	
Law enforcement officer	7
City/district attorney	6
Judge	7
Policy expert	4
Academic researcher	1
Gun violence prevention advocate	2
County of work,^a^ N (%)	
Alameda	2
Contra Costa	1
Los Angeles	4
Nevada	1
Sacramento	4
San Diego	9
San Joaquin	1
San Francisco	1
Santa Clara	3
Outside of California^b^	2
Gender, N (%)	
Male	15
Female	12
Direct GVRO involvement,^c^ N (%)	
Yes	18
No	9

a. One key informant works in two counties.

b. One academic and one policy expert live outside of California but were included for their deep subject matter expertise.

c. Direct involvement includes petitioning for or serving a GVRO, hearing or ordering a GVRO, or providing legal representation for a GVRO petitioner.

### Implementation

Our implementation findings are presented according to the 4 CFIR domains that emerged as most relevant. Table 2 summarizes features of implementation, described below, that encouraged or hindered uptake.

**Table 2:** Contextual considerations for GVRO implementation by CFIR domain and construct.

CFIR Domain	Construct	Barrier	Facilitator
Intervention characteristics	Risk	• Risk of bodily harm during order service and firearm removal	• LEOs serving as petitioners
			• Having agents trained in firearm removal act as servers
			• Obtaining anticipatory search warrants prior to service
	Cost	• Opportunity cost	• LEOs petitioning for emergency GVROs when appropriate
		• Time-intensive process	
	Adaptability	• Resistance by law enforcement to petition for GVROs	• Permitting multiple types of petitioners
Outer setting	Interagency coordination	• Lack of coordination between implementing agencies	• Inter-agency communication and collaboration; co-creation of standards and practices
	Local firearm ideology	• Politicization of the law	• Real-world examples of the law’s utility
Inner setting	Readiness for implementation	• Lack of awareness and education about the law	• Formal training
			• Development of policies and procedures
		• Confusion about roles and responsibilities	
	Culture	• Views regarding the Second Amendment	• Real-world examples of the law’s utility, especially with regard to officer safety
Process	Planning	• No state funding for implementation	• Allocating funding for local implementation
		• No plan for implementation	• Dedicating personnel to coordinate cross-agency implementation procedures
		• Ad hoc procedures	
	Engaging	• Lack of education and training among those responsible for implementation	• In-person training
			• Local champions
			• Prioritization of GVROs by leadership

#### 1. Intervention characteristics

Key informants identified risk, cost, and adaptability as features of the GVRO policy that are important to implementation.

*Risk*. Risk of harm to the respondent, petitioner, or server emerged as a major concern. Given the inherent risk of violence associated with the circumstances giving rise to GVROs, several key informants felt that only LEOs should serve orders and remove firearms, and emphasized that both should be done concurrently, quickly after the order is issued. When firearms are not relinquished at the time of service, the respondent has 24 hours to surrender their firearms, which could provide them time to carry out the original threat, retaliate against the petitioner, or attempt to hide the weapons.

Participants identified respondents’ lack of willingness to voluntarily surrender firearms—which one officer estimated to occur in about 50% of cases—as a critical risk point, often resulting in officers obtaining a warrant. If it is not granted immediately, officers must recontact the respondent who has been alerted to the order, creating a high-risk situation.

Strategies to reduce risk include having only LEOs with specialized training in firearm recovery act as GVRO servers. In the absence of such personnel locally, recovery services can be requested from special agents at the California Department of Justice, who were seen as particularly effective:

*The detectives or special agents that are trained to go out and get the guns are really good at just getting people to just open the door and […] voluntarily and consensually turn in their firearms*. *[…] They show up in their tac[tical] gear […] to make sure that their officers are protected*. *But their demeanor is […] pretty friendly*, *actually*. *And so*, *they’re relatively successful at getting people to just let ’em in and say*, *"Hey*, *you know*, *yeah*, *I got the gun in my drawer–I can go get it*.*”–*Deputy City Attorney (DCA)

To reduce risk at the time of service, key informants also suggested preemptively obtaining an anticipatory search warrant (also called a “trigger” or “contingency” search warrant), which only goes into effect if and when the respondent refuses to cooperate.

*Cost*. Minimizing risk of harm when serving GVROs entails direct and indirect costs, particularly when the respondent fails to voluntarily relinquish their firearms. One key informant highlighted the time and opportunity cost involved in these situations:

*So*, *then the police would have to keep the scene held down*. *Then they have to go obtain a search warrant*. *Then they have to come back and present the search warrant and use the search warrant to go in*. *So*, *that’s a high-risk situation*. *You have to secure a scene*. *You have to surround it*. *You have to take a lot of patrol officers off the street*. *It takes time*. *You*, *of course*, *have alerted the person of what you’re going to do*. *So*, *it’s high-risk and takes a lot of resources*.*–*DCA

Another key informant noted that specialized teams, including professionals outside of law enforcement, can be required in extreme circumstances:

*Then*, *like in some instances*, *we’ve had [the respondents] barricade themselves in and refuse to hand over guns*, *because you’re dealing with people who are unstable*. *Then that’s resulted in [a] SWAT standoff for a few hours and a clinical mental health person engaging in conversation*. *So*, *that has been a hassle and very resource intensive*.*–*DCA

Costs are not limited to risk-mitigation. One LEO described acting as a GVRO petitioner as “very time consuming,” “very personnel intensive,” “court intensive,” and “burdensome.” Another noted that the administrative burden of monitoring GVRO respondents with a local tracking system “eats up personnel hours.” Some officers also expressed concern over lack of adequate time to incorporate GVROs into their workflow. Adding GVRO cases to officers’ existing responsibilities may contribute to lapses in hearing attendance (and the subsequent dismissal of the order), as hearings may involve lengthy wait times and multiple court visits.

To reduce the administrative workload, a key informant suggested that LEOs petition for emergency orders whenever appropriate, as they involve less paperwork than temporary orders and do not require the petitioner to appear in court until the hearing for the 1-year order:

*Make sure throughout the state that the short [emergency] form is allowed to be used by all peace officers filing […] in courts*, *because there’s nothing that makes a cop not want to do something more than sit[ting] in a courthouse for four hours waiting […]*, *filling out paperwork and getting an order*.–LEO

*Adaptability*. While many key informants, particularly LEOs, felt that only law enforcement should act as petitioners to minimize risk, a few noted that allowing others to petition can be critical in parts of the state where law enforcement agencies are more resistant to the law. One expert in firearm legislation noted that the recent expansion of permissible petitioners may be key to implementing GVROs more widely:

*In rural counties […] law enforcement may be antagonistic to taking someone’s guns away under any circumstances*. *We’ve seen in California there has been*, *I believe*, *at least one sheriff who wanted to declare his city a gun sanctuary city*, *so if I were a therapist or anyone living in that town*, *I would not likely feel comfortable going to law enforcement to take someone’s guns away*.

In places where local law enforcement is unable or unwilling to serve a GVRO, an advocate suggested that state agents could step in: “I think that’s where DOJ can really assist in the rural counties to make sure that things are done equally, equitably throughout the state and some of those agencies might not have the ability to go enforce it.” Such adaptability may be particularly important in states like Colorado, where multiple county sheriffs claim they would refuse to petition for or enforce ERPOs [24].

#### 2. Outer setting

Two constructs emerged relating to the external context of organizations implementing GVROs: interagency coordination and local firearm ideology.

*Interagency coordination*. Key informants emphasized the importance of synchronizing efforts across law enforcement agencies, city and district attorneys, and judicial officers to share responsibilities and improve efficiency in implementing GVROs. For example, city attorneys can represent LEO petitioners in court, alleviating some of the burden for law enforcement. They can also provide guidance to LEOs on appropriate use; according to one officer, “…there is one city attorney in [_____] who handles these, and I can run it by [her] and say, ‘Hey, does this meet criteria for gun violence restraining orders? Is it something that would be basically beneficial?’” District attorneys (DAs) also play a role by, for example, referring potential GVRO cases to law enforcement agencies.

Increased coordination between the courts and other implementing agencies was cited as a way to improve implementation. One judge stressed the importance of prosecutors and judges being “on the same page” about standards and practices relating to GVRO cases “just so that we all know, ‘This is how it’s going to look to you.’” A firearm policy expert also suggested that courts could implement scheduling processes that better facilitate participation of respondents and petitioners (including law enforcement), such as having designated time slots for GVROs on their calendars.

*Local firearm ideology*. Some key informants noted problems stemming from the polarization of GVROs and firearm policy more generally: “The standard right now is–the current climate in America, whenever there’s an active shooter, or there’s a school shooting or something like that, is both sides dig their heels so far in they can’t look to reason a lot of times” (LEO). This may have influenced implementation, which was perceived to be tied to local ideology regarding firearm ownership and the Second Amendment. For example, one advocate said that in her “conservative” county, her “Sheriff was very pro-gun and didn’t express interest” in GVROs. In these places, GVROs may be regarded as “liberal” “gun grabs” that are perceived to be threatening Second Amendment rights (DCA).

Grounding descriptions of GVROs in case examples may lessen this politicization. An academic expert on firearm violence explained that after presenting on the policy and its use, “people would come up to me afterwards and say, ‘You know, I came here to disagree with you, but what you say makes sense. You’re not focusing on guns. You’re talking about people. And we all know that there are times in our lives when we just shouldn’t have guns.’” She continued, “I have absolute confidence that I can go to the N[ational] R[ifle] A[ssociation] convention and pull out like a random sample of people. And I can’t imagine that anybody would disagree that if I read the facts of these cases, that they shouldn’t have a gun.”

#### 3. Inner setting

Readiness (i.e., the leadership, education, and resources needed to successfully implement the intervention) and the culture within implementing organizations emerged as key inner setting constructs pertinent to implementation.

*Readiness for implementation*. Key informants agreed that major barriers to the early use of California’s GVRO law included insufficient awareness of the law, knowledge about how or when to use it, and understanding of what was necessary to see an order through. Initial confusion about implementation permeated all involved institutions. As one LEO put it, “In the beginning, there w[ere] a lot of growing pains with it.”

One unique feature of GVROs is that LEOs serve as petitioners. This entails appearing in court and possibly representing themselves (like respondents, who are not provided an attorney in civil court). Counties and municipalities have attorneys to represent law enforcement (e.g., city attorneys or private council on contract), but local considerations inform whether they represent LEOs at GVRO hearings. Without an attorney, LEOs have found hearings difficult to navigate:

*Not having an attorney there and not having an attorney involved in the process has really not made my people happy*. *[…] You’re like*, *“You know*, *yeah*, *it’s easy for the attorneys because you went to law school*.*” […] My job isn’t to debate with some other attorney on whether or not*, *you know*, *the case would be continued*, *or if I turn over the right evidence*, *and how [to]*, *you know*, *subpoena somebody into court*. *You know*, *these are the sort of things that are completely foreign to us*.*–*LEO

Another source of uncertainty was that GVROs are civil orders but are in the penal code with criminal offenses. This was initially jarring for both LEO petitioners and, in some jurisdictions, their legal representatives:

*Just having city attorneys do these as representing cops legally is great*, *but most police officers have no idea who their city attorney is or who their legal rep is*. *[…] And a lot of jurisdictions freak out ’cause cops say*, *"I don’t do civil work*. *I do criminal work*.*" And they’re correct*, *but now they do*.*[…] And then civil attorneys say*, *"This is criminal*. *This isn’t civil*. *I’m not a prosecutor*, *and this is something a prosecutor should do*.*" So*, *everyone feels a little out of their wheelhouse[…] when they first look at this*. *And it can be scary*.*–*DCA

It also created confusion in the judicial system with regard to who should be handling these cases and where they should be routed internally. This has resulted in inefficiencies and delays from officers being “bounc[ed] back and forth” between family and criminal court (DCA).

Formal training on GVRO use, beyond informational bulletins, was recommended to counter confusion about how GVROs should be handled, but the scale of such an effort presents practical difficulties. As one DCA noted, “doing small groups of in-person training, where we can use specific case studies and show them, ‘Hey, this would be a great tool for you,’ that would be most effective. But to get the thousands and thousands and thousands of officers, I don’t know how we would do it.”

In addition, a LEO recommended the development of “strong policy and procedures that makes it easy,” followed by training and monitoring of officer compliance to ensure accountability. The officer suggested creating a field resource that is “basically a threat assessment checklist” that would include petitioning for and serving a GVRO when appropriate. This checklist could complement another officer’s suggestion for formal threat assessment training for LEOs, many of whom are currently “winging [it].”

*Culture*. Generally, participants felt law enforcement to be a conservative and “fairly Second Amendment friendly, gun friendly, gun ownership friendly” group (DCA). One LEO explained, “we have 2,000 cops and 2,000 of us protect the Second Amendment.” A few attested that this may create hesitation to use GVROs. For example, “when you tell the officers that they can remove a firearm without arresting someone for a crime, they kind of feel, like, ‘Well, I wouldn’t want someone to do that to me.’”

As described above in relation to external cultural barriers, key informants suggested that internal cultural barriers could be reduced by providing officers with clear examples of situations in which a GVRO would be useful. For example, in one jurisdiction “there was some resistance to accept this [GVRO policy] until we ha[d] a big shooting and people realize[d] that that particular shooting could have been with our officers, could have been avoided had we been able to go in and get the guy’s weapons” (LEO).

#### 4. Implementation process

Two constructs emerged as key to the implementation process: 1) the degree and quality of advanced planning, and 2) engaging the right people, such as opinion leaders.

*Planning*. Some participants suggested that the lack of education and training within agencies tasked with implementing GVROs was due to the state legislature enacting the law without funding its implementation. As a result, “no one really took notice of it […] because we [were] not given the money to really market it” (advocate). As one LEO detailed, not having a centralized plan guiding its rollout led to widespread confusion about the law and its intended implementation: “It was kind of like the state of California pushed out a law. […] And now, and ‘why aren’t you guys doing it?’ Well, because […] you threw this whimsical idea out there […] and we’re like, ‘we don’t even know what the hell to do with these things,’ right?”

Without a guide for implementation, agencies reportedly took an unplanned “ad hoc” approach that varied across jurisdictions (judge). This, coupled with the fact that GVROs are relatively rare, caused uncertainty about the implementation process. A judge described it in the following way:

*So*, *[a GVRO] gets filed*, *and then where does that go? Who is ruling on them? Where is the hearing held? I would talk to five different bench officers and get five different answers*. *[…] Even internally*, *to the court*, *I think there was a lot of misunderstanding and uncertainty about*, *‘What is our internal process for who’s ruling on [GVROs]?’*

Certain areas engaged in earlier uptake of the law and development of local procedures. The San Diego City Attorney tasked a few of her deputies with investigating how their office could help prevent firearm violence. After identifying the GVRO law as a promising solution, the City Attorney’s office worked with law enforcement agencies and judicial officers to create an integrated process for GVRO implementation in their city, resulting in increased uptake of the law. This process, as described by one of the DCAs involved, is presented in Box 2.

**Box 2. Deputy city attorney on innovating implementation procedures**

*There [were] a couple motivations [to build a GVRO implementation process from scratch]*. *One was a very serious officer involved shooting down here where a GVRO would’ve made a huge difference*. *It made national news*, *I believe*. *[…] Two officers were shot*. *The suspect was killed*. *It’s on YouTube*, *actually*, *the video*. *We show it in our training*. *But that was kind of a big push for us because the city attorney*, *_____*, *after those kinds of situations and also in her own personal experience–she went to U[niversity of]C[alifornia] Santa Barbara*, *where there was a shooting*. *[…] She tasked her office with saying*, *"Hey*, *look through the codes*, *and see if there’s anything we can do*,*" once she got elected*.

*So we found this GVRO law that had been passed in 2016*. *This happened in mid-2017*. *[…] And so what happened was we said*, *"Hey*, *_____*. *[…] Here’s one that looks like it applies*. *I’ve never even heard of this penal code section*, *but it’s here*.*" And the cops can file these*, *and we can take guns away from people who we fear dangerous*, *absent any crime*. *So she met with the chief of police and said*, *"Hey*, *we want to actually file these on people that you think are dangerous*.*" […]*

*So the chief of police and [the City Attorney] met*. *And they agreed*, *yeah*, *let’s try to put this together*. *So we looked up all the forms […]–we met with the courts and said*, *"We want to start filing these*. *What do you think*?*" They were kind of like*, *"Well*, *the rules are the rules*, *and the forms are in there on our page*. *File one*, *and let’s see how it goes*.*" So they came up with their policies and tightened them up knowing these were coming*.

*I worked with the police*. *I worked with my boss […]*. *We formed our own procedures*. *We all met and conferred to make sure there [were] no holes*, *no situations we weren’t accounting for*. *And then we just started doing one case at a time*. *And the first few were really rough*. *No one knew what was going on or what to do*. *We were just following the penal code*. *And then the second one was a little easier*, *third one a little easier*. *And here we are 300 later*.

*Engaging*. San Diego overcame challenges to implementation through the leadership of its City Attorney, a local champion of the law who developed a cross-agency implementation strategy in her jurisdiction. After this strategy was in place, she “started training [agencies outside San Diego] on her own budget because she felt it was a lifesaving tool throughout California” (advocate). In 2018, the California legislature funded her office to train frontline stakeholders across California. One policy analyst warned that this cannot replace jurisdiction-specific training though, as “there’s very little that is consistent around the state in each of the other 57 counties that allow for easy replication.”

Key informants recognized the importance of local champions to successful implementation: “[There is a] need for information about the policy to get to the right people, but we also need […] those local champions to kind of stand up and make it happen” (academic). Prioritization of GVROs by leadership within law enforcement agencies was identified as important to success, particularly when barriers are created by local firearm ideology and the culture within law enforcement. As a DCA put it, we “need a top-down mechanism in law enforcement agencies so that the officers know it is a priority.”

### Outcomes and effectiveness

Key informants reported a range of direct and indirect consequences stemming from GVROs (Box 3 provides specific examples). Direct consequences included increased firearm safety in the form of removing a firearm from a high-risk situation—the primary intervention. In some circumstances, petitioners did not pursue an order after a hearing when respondents attended firearm safety training and began safely storing their firearms. Firearm safety, in turn, may have prevented firearm violence from occurring and in some cases allowed family members of chronically ill individuals to resume caregiving.

**Box 3. Case outcomes**

Deputy City Attorney:

*He was gonna shoot up an entire garage full of people that were servicing his car because he believed they were gonna kill him when he walked in there*. *But he also knew enough about his diagnosis to know that the feelings he was having were wrong*. *So he calls the police on himself*. *They intervene right there on the spot when he’s got the gun in his hand walking into the garage*.

*He says*, *“I’m sorry*. *I just can’t help myself*. *But I know it’s wrong*.*” And he is diagnosed with P[ost] T[raumatic] S[tress]D[isorder]*, *military combat vet*, *the whole deal*. *He let us know afterwards*, *“Thank you*. *Your gun violence restraining order*, *it helped put me on the right track*. *It got me back into the V[eterans] A[ffairs]*. *It got me on some different medicine*.*” And he agreed he shouldn’t have guns*, *and he said if someone had just pulled his guns away sooner*, *we might not’ve gotten to that fracture point that he got to*, *where it was damn near another national shooting*.

Law Enforcement Officer:

*We had another recent one where a guy was being fired from a hospital and he was going back to his house to acquire his weapon and […]we were able to get an emergency GVRO prior to him getting there to set up surveillance on him and stopped him right as he got home before he drove down*. *Actually*, *he was driving back to the hospital and we did a traffic stop on him with the GVRO*. *So we were able to stop him and bring him back to his house and take his weapons*.

*[*…*]*

*We’ve also had several Alzheimer’s patients that have had firearms that we’ve taken and because we’ve been able to utilize our psych teams we’ve been able to facilitate treatment for some of these people*, *or at least more family involvement[…]*. *A lot of the family was terrified of going over there but once we were able to remove the weapons […] then they’re able to actually actively assist this person*.

Judge:

*What I do know*, *and what I am convinced of*, *is the mere exercise of regularly set gun compliance hearings*, *and ordering people upon pain of*, *“I told you about this hearing*. *If you don’t come*, *I’m going to issue a bench warrant for your arrest if you fail to appear*,*” I think the mere exercise of doing that*, *I believe*, *has gotten guns turned in that wouldn’t otherwise be turned in*, *or at least what I can say more confidently is I know that it has gotten proof of turning in guns at a higher rate than they haven’t turned in*, *so I know that*.

Indirect consequences primarily consisted of the respondent accessing needed resources or services. Outside of court, GVROs have led to psychiatric assessments and treatment, and to substance use treatment or rehabilitation programs, including ongoing outpatient therapy. For veterans, GVROs have resulted in connection with Veterans Affairs and other special resources, such as member-assistance drinking programs.

While many participants touted the benefits of GVROs, one noted the potential for the law to reinforce socioeconomic and racial inequities. A firearm policy expert explained (Box 4) that GVROs being civil orders can be a double-edged sword: although civil procedures offer an alternative to arrest and incarceration, respondents are not entitled to legal representation in civil court and violation of an order can lead to criminal penalties. As such, wealthier respondents with means to hire an attorney may receive what they see as favorable outcomes more often than respondents without such means. The same expert expressed concern about GVROs being “misused or underutilized” in communities of color:

*Racism*, *racial bias creates limited access to police as a protective resource*. *[…] One reason [people] in communities of color aren’t calling the police isn’t because they don’t need protection but that’s not protective if [the police are] going to shoot their family members or community members*.

**Box 4. A policy expert on GVROs’ potential to reinforce systemic inequities**

*What I appreciate about GVROs is that they are a civil remedy that provide an option that doesn’t involve incarceration*, *initially at least*, *so that if someone–it’s not appropriate to pursue criminal sanctions*, *probation*, *incarceration and all the consequences that go with a misdemeanor or a felony and all that’s really needed is the removal of the firearms to be protective of themselves or the community*, *then there’s a lot of advantages to that because it can reduce unnecessary incarceration*.

*At the same time*, *my concern is that because law enforcement can obtain these and there are criminal consequences for violation*, *it still pulls people into a state process*. *And because it’s civil*, *they’re not entitled to representation so they can’t get information*, *which I think is a huge access-to-justice issue because we are removing firearms from people who might need them for a job or for other practical necessities*. *And they have the opportunity to make their case*, *but we don’t have attorneys trained to represent them and they’re expensive*. *And most people who are on civil restraining order calendars are self-represented*, *the vast majority*. *And so I am concerned that it’s an uneven playing field when you have essentially the state in the form of law enforcement represented by taxpayer-funded city attorney or county council or another attorney paid for by taxpayers*. *And on the other side*, *you have a self-represented litigant*. *That’s concerning so we’ve already decided we wouldn’t let that happen in a criminal context*. *We would provide representation*.

*So I think it can replicate some of those inequities that put people at a disadvantage*. *In some of the cases I’ve seen wealthier people hire attorneys and are able to work [it] out*. *Maybe they don’t go to a hearing*. *Maybe they just agree to give someone the firearms or remove them so you see that kind of issue*.

#### Evidence strength and quality

Overall, key informants perceived the evidence in favor of GVROs to be compelling but incomplete. In response to the statement, “Gun violence restraining orders are effective at reducing firearm violence,” 5 of 13 key informants strongly agreed and 8 agreed. Two of those who strongly agreed clarified that they perceived GVROs to be effective at preventing firearm suicide, noting support for this in the scientific literature. Almost all of those who endorsed their effectiveness noted that the law is intuitively promising, but that more research is needed: “Anecdotally and intuitively GVROs seem to be effective when used. Of course, it is hard to know what has been prevented” (advocate).

In response to the statement “Gun violence restraining orders save lives and other states should adopt GVRO policies,” 13 of 16 key informants strongly agreed, 2 agreed, and 1 was not sure. Those who strongly agreed tended to stress the utility of GVROs as a tool for law enforcement that fills an important policy gap. As one advocate said:

*There are times when a person is in a dangerous state […] and should not have access to a firearm*. *My daughter was killed by a person who was severely mentally ill in 2001*. *The shooter’s family*, *girlfriend*, *and case worker were concerned about him*. *They knew he had guns*. *Without a GVRO*, *they had no legal tool to remove his firearms*. *[…] In my small town*, *an elderly man with dementia shot and killed his caregiver*. *In my broader community*, *I know 4 people who died by suicide with a firearm*. *Removing access to firearms in these situations could save lives*.

A DCA concurred: “Without a [GVRO] law, there is not a mechanism to provide a cooling off period or provide information to a court about the dangers that ordinary people see.” A LEO strongly agreed because “it gives law enforcement the opportunity to intercede before a crime or a violent act occurs. And in circumstances where they may not be able to use any other legal mechanism.”

Three informants who did not strongly agree with the statement cited the need for data on both long-term outcomes and uptake of the law, but noted that the law is promising. Even without data, they felt, as one Deputy DA put it, “other states should adopt GVRO polices to provide families a tool to help a loved one from hurting themselves or others.” One LEO was more circumspect: “basically being so new, I still need to evaluate, you know, whether there’s a real benefit or not.” He noted the paradox of prevention and the difficulty of such an evaluation: “it’s one of those things where if we prevent something from occurring you may never know that–you may never know, because it was successful.”

## Discussion

California was the first state to enact a GVRO law, but uptake was initially very slow [9]. Using data from semi-structured interviews with key informants, this study identified factors that help explain this limited use and present opportunities to support implementation in California and elsewhere. These factors fell into 4 CFIR domains: intervention characteristics (risk, cost, adaptability), outer setting (interagency coordination, local firearm ideology), inner setting (readiness for implementation, culture), and implementation process (planning, engaging). Key informants overwhelmingly believed the law to be a useful tool for violence prevention, echoing existing data supporting these orders’ effectiveness in preventing suicide [5–7].

The underlying cause of many barriers to implementation in California appeared to be lack of funding for local implementation planning and training, resulting in the absence of standardized processes. Ad hoc practices were adopted in response to orders moving through a system that was ill prepared for processing them, creating variation across the state. Areas with the most robust implementation, such as San Diego, allocated local resources to create specialized teams that developed policies and procedures for GVROs “from the ground up” (DCA) and provided training to LEOs charged with enforcing this law.

Previous studies of ERPO implementation in other states identified many of the same themes we noted here. Single-county studies in Florida and Indiana both found that rollout could be enhanced with early development of implementation protocols and education efforts, both within and across agencies [6, 11]. Without this, uptake of the law was delayed in California, Indiana, Maryland, and Connecticut because of confusion among frontline professionals charged with implementing the orders [5, 6, 25]. Additionally, key informants in Marion County, Indiana and physicians in Maryland (permissible petitioners in that state) noted the labor-intensive nature of petitioning for GVROs [6, 25], as did several of our participants.

Our key informants overwhelmingly supported the law and believed it to prevent firearm violence, suggesting that those most familiar with utilizing the law are likely to favor it. They noted a range of benefits to respondents, extending beyond firearm violence prevention to include extrajudicial remedies of substance use treatment, mental health care, and medication. However, there is potential for inequitable distribution of these benefits, as wealthy respondents may have access to legal representation unavailable to others, and people of color may be reluctant to voluntarily involve their loved ones in the legal system or engage with law enforcement out of fear of being disenfranchised, brutalized, or killed. Ensuring that all counties offer GVRO-specific legal assistance for self-represented respondents and petitioners—particularly family members and other non-LEO petitioners who are most likely to be unfamiliar with the process—would be an excellent first step in addressing this issue.

We offer several recommendations for improving implementation and uptake of GVROs. For states that have adopted but not yet implemented an extreme risk law, we suggest legislatures appropriate funds supporting local implementation statewide. This would include localities creating procedures for implementation and providing training prior to when the law goes into effect. We recommend that legislators in California allocate funding for training on GVROs and other orders that mandate firearm removal (e.g., domestic violence restraining orders) to city attorneys, judges, and LEOs, and integrate these orders into basic law enforcement training on firearm relinquishment. Where needed, this would necessitate the specification of roles and responsibilities within each agency and the formulation of local policies and procedures for implementation. In 2019, the California legislature considered Assembly Bill-165, which would incorporate GVRO education into LEO basic training. This measure should be adopted and similar training should be provided to established LEOs, city attorneys, and judicial officers.

We also recommend that law enforcement agencies consider having officers specifically trained in firearm removal serve orders. Having such personnel would allow for specialization that would save time and reduce risk of violence. In addition, law enforcement and city attorneys should collaborate in a manner appropriate to local conditions to provide LEO petitioners with legal representation whenever possible.

We further suggest that the judiciary include a unit on GVROs during judicial training for civil court judges that may hear these cases. All new judges in California attend “Judicial College,” a mandatory 2-week training course that covers a range of topics. Adding GVRO training to this program would support increased judicial awareness of GVROs and standardization of practices across jurisdictions.

Finally, we recommend funding and data be made available to researchers to study ERPO processes and outcomes. More research is needed to understand whether and in what circumstances ERPOs reduce firearm violence; how the equitable distribution of ERPOs and other temporary firearm transfer strategies can best be promoted; and what respondents experience. This research can, in turn, inform the targeted and equitable uptake of the law.

### Limitations

Our key informants represent a small number of counties, all of which have issued GVROs, and were likely more supportive of GVROs than their counterparts who have not implemented the law. This prevented us from learning why some jurisdictions have not implemented GVROs but enabled us to have a fuller understanding of implementation in areas with the greatest use. Additionally, we were unable to contact petitioners who were not LEOs, such as family members. Findings may not represent the perceptions or experiences of this group. While non-LEO petitioners were rare, accounting for only 3.5% of petitioners from 2016 to 2019 [9], their insights and experiences of the process would be extremely valuable. Future research should prioritize reaching this group.

Due to the semi-structured nature of the interviews, not all key informants were asked the Likert scale questions about the law’s effectiveness. Those who were not asked might have answered differently than those who were. Finally, it is possible that the interviewers unintentionally influenced participants’ responses, though having trained, experienced interviewers minimized this possibility.

## Conclusions

ERPOs fill a critical gap in firearm policy. These laws have the potential to prevent firearm violence, but as participants emphasized, more research is needed to fully understand the law’s impact. Key informants, including law enforcement, attorneys, judges, and firearm violence prevention experts, indicated that the law’s rollout in California was troubled but that improvements are possible with local leadership, collaborative policies and procedures, and statewide education efforts. Many states are or will be implementing ERPO laws; we hope that the advice of these experts can guide their efforts.

## Supporting information

S1 Appendix Interview protocols. (PDF)

## Abbreviations

CFIR: Consolidated Framework for Implementation Research
DA: District Attorney
DCA: Deputy City Attorney
ERPO: extreme risk protection order
GVRO: gun violence restraining order
LEO: law enforcement officer
