# Supplementary material for: Implementation and perceived effectiveness of gun violence restraining orders in California: A qualitative evaluation
Source: PLoS One. 2021 Oct 19;16(10):e0258547. doi: 10.1371/journal.pone.0258547 (PMC8525775; doi:10.1371/journal.pone.0258547)
Supplement: S1 Appendix — (PDF) [file pone.0258547.s001.pdf]

Hello Mr./Ms. ________. My name is ________ and I'm calling about the gun violence restraining order (GVRO) process here in California. As you may know, the Violence Prevention Research Program at UC Davis is working with the California Department of Justice on an assessment of GVROs. We are examining the implementation of the policy and evaluating the effectiveness of the orders in preventing violence. We identified you as a key informant for our assessment, based on your experience with the implementation of GVROs. Your expertise and insights will help us better understand GVROs and their effects and will inform further implementation efforts. Any information you share will remain anonymous unless you give us your permission to disclose it with attribution.

May we proceed with the interview?

If NO, can't talk now: Is there a better time for me to follow-up instead? Can we schedule something now?

If YES, obtain informed consent.

Note: text in red can be skipped for judges. Text in blue is for judges only.

1) Overview of the GVRO policy
   a) Can you explain to me the purpose of a GVRO, as you understand it?
   b) **For what kinds of cases do you think GVROs are intended? For which do you think they are most suitable?**
   c) *(For select key informants)*: We have an idea about how GVROs work, but we'd like to hear your perspective. Would you be willing to review our summary model of why we might expect GVROs to work for some people in some contexts via email and provide feedback? *If YES:* What address should I email it to?

Throughout these next topics, please distinguish between emergency orders, temporary orders, and orders after a hearing where that would be important.

2) Professional involvement in GVRO enforcement
   a) **Approximately how many GVRO cases have involved you personally, or have involved your agency or organization?**
   b) **What role did you or your organization play in these cases**?
      i) Petitioner? Server? Legal representative? Judge?
      ii) How is it determined who in the organization will take on GVRO cases?

Thinking about the cases you (or your organization) have been involved with:
   c) **What circumstances led to the decision to petition for GVROs in these cases?** How have emergency and temporary orders differed, in your experience?
      i) *Encourage interviewee to recount specifics of cases*
   d) Where did the key information come from? Family? Other civilians? Law enforcement professionals?
   e) **What alternatives were considered, if any, and why was a GVRO chosen?**

f) **Was the specific intent of the GVROs to recover firearms already in the respondent's possession, to prevent known purchases that were in process, both, or something else?**

3) GVRO process: petitioning
   a) Describe, from your point of view, the process of petitioning for a GVRO.
   b) Do you think the necessary information available and easy to access?
   c) Do you have any recommendations for improvement?

4) GVRO process: court approval/denials
   Not judges:
   a) **Have you had a petition denied?**
      i) What were the specifics of the case and reasons for denial?
      ii) Did you feel the denial was justified?
      iii) Are you aware of what happened with the respondent after the petition was denied?
   Judges only:
   b) **Can you explain your decision-making process when you are determining whether or approve or deny a GVRO petition?** What do you take into consideration and how do you weigh pros and cons?
      i) Does this differ with the type of GVRO at issue? In what way?
   c) **Have you ever denied a petition?**
      i) What were the specifics of the case and reasons for denial?
      ii) Are you aware of what happened to the respondent after the petition was denied?

5) GVRO process: service/non-service and firearm recovery
   a) **Describe, from your point of view, the process of serving a GVRO.**
      i) Have you personally served a GVRO? What was that like?
   b) Are you aware of any GVROs being served by someone other than law enforcement?
   c) **Are you aware of there being unanticipated events while an order was being served or when firearms were being recovered?**
      i) Has anyone been threatened or hurt?
   d) ***Not judges:* Have you had orders granted but not served?**
      ***Judges only:* Have you ever granted an order that was not subsequently served?**
      i) What were the specifics of the case and reasons for non-service?
      ii) Are you aware of what happened to the respondent since the petition was not served?
   e) Thinking about the cases you are familiar with: **how frequently, when firearm and ammunition recovery was the objective, was recovery achieved?** How often are all firearms and ammunition recovered?
   f) When a GVRO has been initiated after law enforcement makes contact with an individual in the field, does law enforcement ever take temporary custody of firearms/ammo prior to obtaining a GVRO?
   g) **Do you have any recommendations for how the service or recovery process could be improved?**

6) GVRO process: post-service experience

a) Describe, from your point of view, what happens after GVROs are served and firearms are recovered.
   i) What is the ideal scenario? How does this differ from reality, in your experience?
b) Thinking of the cases you are familiar with: **are you aware of specific beneficial or adverse consequences that resulted from the GVRO?**
c) **In your experience, how often are emergency or temporary orders followed by orders after hearings?**
d) Have you been involved in hearings to extend a GVRO for up to a year?
   i) What was that like?
   ii) What kind of evidence is provided? What is taken into consideration?
e) When a temporary order is not followed by an order after a hearing, what circumstances make that appropriate—or not appropriate—in your judgment?
f) ***Judges only:*** **Have you ever had a subject petition to terminate a GVRO?**
g) *Law enforcement only:* **How often have subjects elected to surrender firearms to a local law enforcement agency or sell/store them with a licensed firearms dealer, instead of surrendering them to the law enforcement officer serving the GVRO petition?** What are your thoughts on these instances?
h) In your experience, **how often are recovered firearms returned to respondents**?
i) What else occurs after orders are no longer in effect?
j) Thinking about the cases you are familiar with: **what adverse consequences have occurred, if any, after orders are no longer in effect?**
k) **Do you have any recommendations** for how the post-service period could be improved?

7) Opinions on the GVRO policy
a) How much would you agree with the following statement: “Gun violence restraining orders save lives, and other states should adopt GVRO policies.” Would you say you strongly agree, agree, disagree, strongly disagree, or aren’t sure if you agree or disagree?
   i) Why?
b) In your view, **what measures are appropriate in assessing the effectiveness of the GVRO policy?**
c) **How much would you agree with the following statement: “Gun violence restraining orders are effective at reducing firearm violence.” Would you say you strongly agree, agree, disagree, strongly disagree, or aren’t sure if you agree or disagree?**
   i) Does your assessment differ for different outcomes, such as interpersonal violence, mass violence, and suicide?
   ii) In your experience, what has been the response of the parties to the GVRO cases to the GVRO policy?
d) A lot of people have concerns about inequalities in who the criminal justice system polices, arrests, charges, and formally sanctions, particularly with regard to race and socioeconomic status. Do you think this is an issue with GVROs?
   i) Why or why not?
e) GVROs were designed to be implemented using existing law enforcement and court personnel and resources. How well prepared do you think these systems are for taking on GVROs?
   i) What is your experience with this?

f) We know that some counties have been quicker to make use of GVROs than others, but we don't know why. What do you think accounts for this difference in uptake?
g) **GVROs may be requested by members of a respondent's family or household, or by law enforcement. Yet, most GVROs in California involve law enforcement petitioners. Why do you think this is?**
    i) Do you think this is a good or bad thing? Why?
    ii) Do you have a sense of how often GVRO petitions from law enforcement actually start with a request from a family or household member?
    iii) Do you think this trend will persist in the future? (*Probe in case of confusion:* Who do you think will be petitioning for GVROs in one year? Five years?) Why?
h) **Do you think the GVRO law could be improved? How?**

8) Professional awareness
    a) **How would you describe the state of knowledge and use of GVROs among people in your profession**? How could it be improved?
    b) Have you personally engaged in efforts to increase knowledge and use of GVROs among people in your profession?
        i) *If YES*: What have you done?
        ii) What would you be willing to do in the future?

9) Assistance to our study
    a) Are there documents connected with the cases we have discussed that our team could access for this project?
    b) Do you have other key informants in mind whose expertise would contribute to this assessment of GVROs?
    c) Would you be willing to speak with us about the GVRO policy implementation again as our work continues?
        i) *If YES:* What is the best way to contact you in order to schedule the interview in the future?

10) Interviewee comments
    a) Is there anything I haven't asked about that you'd like to tell me?
    b) Do you have any questions for me?

11) Closing
    a) *Thank the interviewee for participating and for their time.*
    b) *Give the interviewee our contact information*

Hello Mr./Ms. ________. My name is ________ and I'm calling about the gun violence restraining order (GVRO) process here in California. As you know, the Violence Prevention Research Program at UC Davis is working with the California Department of Justice on an assessment of GVROs. We are examining the implementation of the policy and evaluating the effectiveness of the orders in preventing violence. We identified you as a key informant for our assessment, based on your experience with the GVRO policy. Your expertise and information will help us better understand GVROs and their effects and will inform further implementation efforts. Any information you share will remain anonymous unless you give us your permission to disclose it with attribution.

May we proceed with the interview?

If NO, can't talk now: Is there a better time for me to follow-up instead? Can we schedule something now?

If YES, obtain informed consent.

1) Overview of the GVRO policy
   a) Can you explain to me the purpose of a GVRO, as you understand it?
   b) What is your understanding of how and when GVROs work as a tool to prevent violence? **For what kinds of cases do you think GVROs are intended?** For which do you think they are most suitable?
   c) *(For select key informants*): We have an idea about how GVROs work, but we'd like to hear your perspective. Would you be willing to review our summary model of why we might expect GVROs to work for some people in some contexts via email and provide feedback? *If YES:* What address should I email it to?
1) Professional involvement in GVRO policy
   a) **What is your professional involvement in the GVRO law?**
   b) How did you/your organization come to be involved with GVROs?
   c) Have you/your organization been directly involved in any GVRO cases?
      i) How were you involved?
      ii) What circumstances led up to the GVRO?
      iii) What was the outcome in this case?

2) GVRO policy
   a) How much would you agree with the following statement: "Gun violence restraining orders save lives, and other states should adopt GVRO policies." Would you say you strongly agree, agree, disagree, strongly disagree, or aren't sure if you agree or disagree?
      i) Why?
   b) What was the inspiration for the GVRO law? Why did you think it was necessary?
      i) Was the law modeled after anything?
      ii) What was the process of writing the law? Who was involved (directly or indirectly)?
      iii) What changes have been or are being proposed?
         (1) Do you agree with these changes?
      iv) What challenges were there in passing the law?
   c) **What challenges, if any, does the GVRO law currently face?**

i) Opposition?
ii) Public awareness?

d) A lot of people have concerns about inequalities in who the criminal justice system polices, arrests, charges, and formally sanctions, particularly with regard to race and socioeconomic status. Do you think this is an issue with GVROs?
   i) Why or why not?
e) How does the law protect the rights of the respondent?
   i) Is this adequate, in your opinion?
f) GVROs were designed to be implemented using existing law enforcement and court personnel and resources. How well prepared do you think these systems are for taking on GVROs?
g) **In your view, what measures are appropriate in assessing the effectiveness of the GVRO policy?**
h) **How much would you agree with the following statement: "Gun violence restraining orders are effective at reducing firearm violence." Would you say you strongly agree, agree, disagree, strongly disagree, or aren't sure if you agree or disagree?**
   i) Does your assessment differ for different outcomes, such as interpersonal violence, mass violence, and suicide?
i) **We know that some counties have been quicker to make use of GVROs than others, but we don't know why. What do you think accounts for this difference in uptake?**
j) GVROs may be requested by members of a respondent's family or household, or by law enforcement. Yet, most GVROs in California involve law enforcement petitioners. Why do you think this is?
   i) Do you think this is a good or bad thing? Why?
   ii) Do you think this trend will persist in the future? (*Probe in case of confusion:* Who do you think will be petitioning for GVROs in one year? Five years?) Why?
k) **Do you think the GVRO law could be improved? How, specifically?**

3) <u>Public awareness</u>
   a) How would you describe the state of knowledge and use of GVROs among people in California? How could it be improved?
   b) Have you or your organization engaged in efforts to increase knowledge and use of GVROs among people in California?
      i) *If YES*: What have you done?
      ii) What would you be willing to do in the future?

4) <u>Assistance to our study</u>
   a) **Do you have other key informants in mind whose expertise would contribute to this assessment of GVROs?**
   b) Would you be willing to speak with us about the GVRO policy again as our work continues?
      i) *If YES:* What is the best way to contact you in order to schedule the interview in the future?

5) <u>Interviewee comments</u>
   a) Is there anything I haven't asked about that you'd like to tell me?

b) Do you have any questions for me?

6) Closing
   a) *Thank the interviewee for participating and for their time.*
   b) *Give the interviewee our contact information*
